# An online review of informational sources for advanced or high-risk cutaneous squamous cell carcinoma

**DOI:** 10.1007/s00520-021-06034-x

**Published:** 2021-02-08

**Authors:** Rachel Starkings, Valerie Shilling, Lesley Fallowfield

**Affiliations:** Sussex Health Outcomes Research and Education in Cancer (SHORE-C), Brighton and Sussex Medical School, University of Sussex, Brighton, UK

**Keywords:** Cancer, Internet, Online information, Patient education, Skin cancer, Squamous cell carcinoma

## Abstract

**Objective:**

Cutaneous squamous cell carcinoma (cSCC) is one of the most prevalent non-melanoma skin cancers worldwide. While usually treatable, patients with high-risk or advanced disease have few treatment options and limited resources available. This review assesses what online information resources are available to patients and their families about either high-risk or advanced cSCC.

**Methods:**

Searches were run, via Google, using 8 terms such as ‘advanced cutaneous squamous cell carcinoma patient information’. Advertisements were removed and the first 3 pages/30 results from each search were screened for duplicates and then against eligibility criteria. Websites needed to have been updated within the past 5 years, be freely accessible, designed specifically for patients and refer to the advanced disease or high-risk setting. Remaining results were assessed using the DISCERN tool.

**Results:**

Of the final 240 results, 121 were duplicates and 104 were ineligible. The remaining 15 sources were predominantly aimed at American audiences, used variable terminology and revealed differing treatment pathways. Only 3 sites were deemed as ‘high’-quality information sources.

**Conclusion:**

There is a lack of accessible online information on high-risk or advanced cSCC for patients. What is available is often too scientific or clinical and lacks clarity about the disease and treatment options.

**Practice Implications:**

Further work is needed to improve the integrity and accessibility of online sources and to signpost patients to the most reliable information. This should include elements of patient led research, clinical education and information development.

## Introduction

Non-melanoma skin cancer (NMSC) is one of the most prominent cancers globally. Under that classification, cutaneous squamous cell carcinoma (cSCC) is the second most common skin cancer [1, 2]. While rates of skin cancer are increasing, cSCC in particular is growing with an anticipated increase amongst 30–39-year-olds [3–5]. Over 95% of patients will be cured of the disease following surgery, with or without radiotherapy [6, 7]. However, the remaining proportion will progress to advanced disease, known as advanced cSCC. While this is a smaller group of patients, survival drastically reduces to a median of 2 years [8], leaving advanced cSCC as the second most common cause of skin cancer death after melanoma [3]. Some of the best indicators that a patient is more likely to be at ‘high risk’ of progression are disease location and previous local recurrence, but further genomic markers are being developed [9].

Within the advanced disease setting, there are anxieties about further treatment possibilities and survival. The care pathway for cSCC is fairly straightforward; however, treatments in the advanced setting are limited, with some evidence for disease control using systemic therapies and EGFR inhibitors. Following recent treatment advancements, PD-1 inhibitors such as cemiplimab have been authorized for this disease group with further investigations ongoing into other immunotherapy options [6]. Additionally, patients may face concerns over the impact of surgical scars and treatment, along with the appearance of the diseased area [10]. As a high proportion of these cancers appear on the head and neck, there is an increase in anxiety, body image concerns and embarrassment [5, 11]. Although this is a smaller group of patients, this burden indicates a need for clear signposting towards support and resources.

These information needs are one of the most unmet areas of the cancer experience [12]. Within the broader NMSC setting, research has shown that patients are often unsatisfied with information provision, leading to greater dissatisfaction and poorer rates of health related quality of life (HRQoL) [13]. There is a lack of research about this dedicated to the advanced cSCC cohort; however, guidelines stipulate that these patients, or those at high risk, should be provided with information about the disease with access to a multidisciplinary palliative team [14]. Unfortunately, this group of patients is not often offered appropriate psychosocial support or even a clear pathway for treatment [15, 16].

Faced with a dearth of information from the clinical team, patients will seek additional information sources about their condition, including conducting online searches [12]. Estimates range from 16 to 69% of people with cancer using the Internet for information [17]. This then raises concerns about how reliable and consistent information is, further compounded by low population levels of health literacy [17–19]. Good quality information can help inform and empower patients; however, when that material differs from that relayed by the clinical team, the patient may feel less satisfied with their treatment [18, 19].

In this paper, we review the online platforms available to patients when searching for information about high-risk or advanced cSCC. Terms were selected as representative of what patients may look for when going online for this purpose.

## Methods

Our primary aim was to identify the scope of online resources available to individuals searching for information about advanced or high-risk cSCC. A secondary aim was to assess the quality of the resources found.

We carried out online searches using eight terms: ‘advanced cutaneous squamous cell carcinoma’, ‘advanced cutaneous squamous cell carcinoma patient information’, ‘high risk cutaneous squamous cell carcinoma’, ‘high risk cutaneous squamous cell carcinoma patient information’, ‘squamous cell carcinoma’, ‘squamous cell carcinoma patient information’, ‘skin cancer’ and ‘skin cancer patient information’. High-risk and advanced terms were separated out to reflect the different needs of these patient groups. We included the terms ‘squamous cell carcinoma’ and ‘skin cancer’ as terminology can often be muddled or confusing for patients. We planned to search specific websites namely the British Skin Foundation, Cancer Research UK (CRUK) and Macmillan for relevant patient-related information. We included this to capture the two main pathways patients might utilize, either using a search engine or going directly to specific, well known, websites.

Guidelines for conducting the review were documented and agreed to by the research team prior to conducting the searches. This guidance was modelled on methods used by Weeks et al. [20]. Searches were run using Google and the top 30 results, or first three pages, of each were retained. Google was selected for its popularity and general accessibility. Typically, the first few results or the first page of searches is used by individuals looking for information [21]. We extended beyond this to ensure we had a wider understanding of what was available. Advertisements and sponsored results were documented but removed from the main searches due to the variability that can occur based on geography, demographics and Internet search history.

Each search was run on its own separate day between 16 June and 5 August 2020. Internet history was cleared prior to each search to avoid confounding results from previous browsing. The top 30 search results were entered onto an Excel spreadsheet with corresponding name, URL and search position. This was then verified by a second researcher who ran the same term, using Google, on the day of the original search. We aimed to achieve an 85% agreement of sites present in both the original search and validation. We also documented any changes to ordering.

Once searches were complete, they were screened for duplicate entries. A site was considered a duplicate if a URL pointed to the same page, or suite of information, as a previous result, or was a secondary repository for the information. For example, a ResearchGate finding would be considered a duplicate to a link for an academic publication. Similarly, a link to a ‘signs and symptoms’ page would be considered a duplicate to a ‘treatment pathway’ page hosted by the same organization.

The remaining entries were screened against inclusion and exclusion criteria. Our inclusion criteria for this project were that sources should be charitable or health-based online resources for patients and the public, with a focus on either advanced or high-risk cSCC. Sites were excluded if they did not feature the advanced or high-risk disease setting or were older than 5 years, personal blogs or websites, not available in English, clinical or academic materials or behind a log in or paywall.

These processes had a 10% verification by a second researcher to ensure duplicates were marked appropriately and that there was an agreement with inclusion/exclusion.

Those results that met the inclusion criteria were then assessed against the DISCERN tool [22]. This measurement is for individuals to assess the reliability of a health-based publication. It is a standalone tool so does not require fact-checking of the information. Instead, each publication is assessed via 16 questions around areas which are deemed important when considering treatment options, for example shared decision-making. These were completed by one researcher with 50% verification by another to ensure reliability.

As this project did not require human participants, no ethical approval was sought.

## Results

Across the eight search terms, there were a total of 240 results. Of these, 121 were duplicate items and 104 were excluded, leaving a final sample of 15 webpages.

The majority of excluded pages were classified as academic or clinical resources. This was particularly true in connection with the first four, more specific, search terms (‘advanced cutaneous squamous cell carcinoma’, ‘advanced cutaneous squamous cell carcinoma patient information’, ‘high risk cutaneous squamous cell carcinoma’ and ‘high risk cutaneous squamous cell carcinoma patient information’). In comparison, sites rejected in searches 5-8 (‘squamous cell carcinoma’, ‘squamous cell carcinoma patient information’ ‘skin cancer’ and ‘skin cancer patient information’) were done so because of their lack of information about the advanced or high-risk setting. Returns were often rejected for more than one criterion with the overall proportions as follows: 61.5% were clinical or academic, 35.6% did not have information on the advanced setting, 13.5% were not updated within 5 years and 10.6% were behind a paywall or required login details. The search results for terms 5–8 included the more well-known charitable websites, and therefore, we did not need to take the planned additional step of a focused review.

Within the search returns, there was a 96.25% agreement between the two researchers conducting the searches, which translated into 9 differing results. The range of agreement within searches was 87.67–100%. This variability was more present within the broader search terms (searches 5–8), which also featured additional changes to ordering.

The final 15 results (Table 1) composed of six (40%) charitable or health-based organizations, e.g. Macmillan [23–28]; five (33.3%) hospital-based pages, e.g. the Mayo Clinic [29–33]; and four (26.7%) online resources, e.g. Wikipedia [34–37]. Sites were predominantly aimed at American audiences (66.7%) [23–25, 27, 29–32, 34, 35] with only three UK specific pages (20%) [26, 28, 33].

**Table 1:** Content comparison

Resource name	Author	Search position(s) (search, result)	Ad-based result?	Date last updated*	Intended audience inc. country	Treatments mentioned**	Pathways mentioned	Any signposting?	Use of advanced and/or high risk terminology?	Any definitions given?
Skin Cancer: Squamous Cell Carcinoma	American Academy of Dermatology	2, 8 5, 23 6, 14–15 7, 14	No—page supported by Neutrogena	2020	Public in the USA	• Surgery • Radiotherapy • Immunotherapy—cemiplimab	Dermatologist with oncologist in the advanced setting	References given	Advanced	Advanced
Basal and Squamous Cell Skin Cancer	American Cancer Society	1, 15 2, 9 4, 20 5, 6–7 6, 7 7, 3 8, 12–13	No	July, 2019	Patients and the public in the USA	• Lymph node dissection • Chemotherapy • Immunotherapy	Primarily dermatologist, in advanced setting surgeon, oncologist, radiologist with other members of a wider team e.g. nutritionists and social workers	Further references on the ACS website, including PDF downloads of the information	Advanced	No
Skin Cancer (Non-melanoma)	Cancer.Net	7, 13 8, 18	No	July, 2019	Patients in the USA	•Immunotherapy—cemiplimab, pembrolizumab	Not included	Further references given including finances, clinical trials and medical phrases	Advanced and metastatic	No
Squamous Cell Carcinoma	The Christie	5, 21 6, 10 6, 12–13 7, 25 8, 9	No	Dec, 2015***	Patients in the UK, being treated at the Christie	• Surgery • Radiotherapy • Chemotherapy	Head, neck and skin team	Further signposting for radiotherapy information	Metastasize	Metastasize
Skin Cancer	Cancer Research UK	7, 7 8, 29	No—page supported by Dangoor Education	Sept, 2019	Patients in the UK	• Surgery • Radiotherapy • Chemotherapy	GP in the first instance and then reference to seeing a specialist as part of an MDT	Further references given	No	No
Cutaneous Squamous Cell Carcinoma	DermNet NZ	1, 22 2, 4 3, 3 4, 2 5, 5 6, 11	No	Dec, 2015	Clinicians, health professionals and health literate public in New Zealand	• Surgery • Radiotherapy • EGFR inhibitors • Cemiplimab	Multidisciplinary pathways	References include links for further information e.g. BAD	Advanced and metastatic used interchangeably, high risk	Metastasize
Navigating Life with Advanced CSCC	Healthline	1, 10 2, 17 5, 8	No	Feb, 2020	Patients on treatment in the USA	• Surgery • Chemotherapy •Immunotherapy—cemiplimab	Oncologist, dermatologist and surgeon with additional pathways, i.e. massage therapy, palliative care	References provided with signposting to local support groups and the American Cancer Society	Advanced	No
Skin Cancer	Macmillan	5, 28 6, 16 7, 12 8, 8	No	2020	Patients in the UK	• Lymph node dissection • Radiotherapy • Chemotherapy • Clinical trials	GP, Dermatologist, MDT	References given	No	No
Squamous Cell Carcinoma of the Skin	Mayo Clinic	5, 3 6, 4 7, 6 8, 1	No	June, 2019	Patients in the USA	• Chemotherapy • Drug treatments • Immunotherapy	Dermatology, surgery, oncology	References to information within the Mayo Clinic site	Spread	No
Squamous Cell Carcinoma	Memorial Sloan Kettering	5, 16 6, 26	No	2020	Patients in the USA being treated at MSK	• Surgery • Radiotherapy • Clinical trials	Dermatologist, surgeon, plastic surgeon, oncologist, survivorship center/counseling	Related topics given such as sunscreen usage	High risk and metastatic	Metastasize, recurrent, high risk
Squamous Cell Carcinoma	Moffitt Cancer Center	5, 26 6, 19	No	2018	Patients in the USA, being treated at the Moffitt center	• Surgery • Radiotherapy • Chemotherapy • Clinical trials	Dermatologist, oncologist, surgeons, plastic surgeons, radiotherapists, pathologists, supportive care	No	High/low risk and metastatic	Metastatic
Skin Cancer Treatment—Patient Version	National Institutes of Health	6, 23 7, 10 8, 3	No	July, 2020	Patients in the USA	• Surgery •Immunotherapy—cemiplimab • Clinical trial	Not included	Further NCI links	Metastatic	Metastatic
Types of Skin Cancer	NYU Langone	7, 29	No	2020	Patients in the USA	• Lymph node dissection • Reconstruction • Chemotherapy • Clinical trials	Psychological support, nutrition, supportive therapies, rehabilitation	Clinical trial links	Advanced	No
Advanced Squamous Cell Carcinoma Treatment	Skin Cancer Foundation	1, 1 2, 1 4, 28 5, 1–2 6, 5 7, 4 8, 7	No—page is funded by educational grant from Sanofi and Regeneron	May, 2019	Patients in the USA	• Surgery • Radiotherapy •Immunotherapy—cemiplimab	Multidisciplinary team including dermatologist, surgeons and physicians	No	Advanced and metastatic	Advanced, locally advanced, metastatic
Squamous Cell Skin Cancer	Wikipedia	3, 25 4, 19 5, 12 7, 19	No	Apr, 2020	Non-specific	• Surgery • Radiotherapy • Adjuvant therapy	Not included	Signposting to DermNet NZ and the Skin Cancer Foundation	High-risk	No

*Multiple dates may be provided over the suite of information on a website. For consistency, when multiple dates are given, that listed here is from the overview page

**Treatments listed here are those specified for the high-risk or advanced setting. Other treatments may be included on the webpage for CSCC

***An update has been made to this page since 2015 but is not yet available online

Most results came through multiple times across the various search terms; however, pages such as the American Cancer Society [27], DermNet NZ [36] and the Skin Cancer Foundation [24] had broad coverage irrespective of the specificity of the search term.

The included results varied in their use of ‘advanced’, ‘metastatic’ or ‘high risk’ language. There were 11 sites (73.3%) that used the terminology of advanced disease [23–25, 27, 29, 30, 32–36] and four that used ‘high risk’ (26.7%) [29, 30, 36, 37]. Two sites did not use any terminology (13.3%) [26, 28]. Sometimes, these words were not explicitly used, for example metastatic disease was characterized by the word ‘spread’ [31]. More often, these terms were used without a clear definition of what they meant.

There were discrepancies in how sites referred to diagnosis and treatment pathways; some did not specify who might be involved in care [25, 34, 37], some started with a physician or general practitioner (GP) and then outlined wider specialists who may be involved, such as a dermatologist [26, 28], while others gave further details on a multidisciplinary team (MDT) including supportive and palliative care [23, 24, 27, 29–33, 35, 36].

There was further variability in the treatments mentioned. Following the advent of cemiplimab in 2018, six pages mentioned it specifically (40%) [23–25, 34–36], with another two discussing immunotherapy more broadly (13.3%) [27, 31]. None of the UK specific websites talked about immunotherapy. Five of the webpages discussed participating in clinical trials (33.3%) [25, 26, 29, 30, 32]. Most pages included more comprehensive coverage of surgical techniques, relevant to patients with or without advanced or high-risk disease.

Of the 15 DISCERN scores, four sites were rated as ‘low’ (26.7%) [24, 29, 30, 33], eight were rated as ‘moderate’ (53.3%) [23, 25, 26, 28, 31, 32, 35, 37] and three were rated as ‘high’ (20%) [27, 34, 36] (Table 2). There were notable inconsistencies across the pages. While some emphasized the ability, and the importance, of shared decision-making, others did not explicitly encourage a discussion between patient and physician. While no website favored one treatment over another, some did not include side effects to treatment or even explicitly name the benefits. Notably absent from all those but the highest scoring pages was a discussion about the impact on quality of life from treatment, including the option of not having treatment. Similarly, those pages that scored lower did not discuss any areas of uncertainty.

**Table 2:** DISCERN quality assessments

Resource name	Author	Overall DISCERN score	Higher scoring points	Lower scoring points
Skin Cancer: Squamous Cell Carcinoma	American Academy of Dermatology	Moderate	• Clear references • Relevant information • Discussion of different treatment types	• Lacking discussion about shared decision-making • No discussion about no treatment • Limited reference to areas of uncertainty
Basal and Squamous Cell Skin Cancer	American Cancer Society	High	• Support for shared decision-making • Information is well-balanced • Relevant information • Clear references	• Little discussion about the impact of treatment on QoL
Skin Cancer	Cancer.Net	High	• Clear layout and signposting • References shared decision-making • Includes information on palliative care • Good description of treatments	• No references • No discussion about no treatment
Squamous Cell Carcinoma	The Christie	Low	• Clear layout of sections • Provides an overview of treatment	• No specifics about treatment including benefits and risks • No discussion about shared decision-making • Not aimed at an advanced audience • No references given
Skin Cancer	Cancer Research UK	Moderate	• Provides description of each treatment • Offers side effects of each treatment • Provides references	• No discussion of newer treatments • No discussion about uncertainty or impact on QoL • Does not talk about shared decision-making
Cutaneous Squamous Cell Carcinoma	DermNet NZ	High	• Clear references and signposts • Description of how treatments work • Discussion of risks and some benefits to treatments • Well-balanced and unbalanced	• No discussion about uncertainty or impact on QoL
Navigating Life with Advanced CSCC	Healthline	Moderate	• Well balanced information • Focus on multiple treatments • Some reference to QoL	• Limited reference to uncertainty • Could elaborate on treatment side effects and benefits
Skin Cancer	Macmillan	Moderate	• Additional signposts provided • Some discussion about body image • Treatments are well balanced	• Does not discuss newer treatments • Does not explain risks with treatment • No discussion about uncertainty
Squamous Cell Carcinoma of the Skin	Mayo Clinic	Moderate	• Signposts and references provided • Clearly laid out • Balanced treatment discussions	• Does not discuss risks or no treatment • Does not reference newer treatments
Squamous Cell Carcinoma	Memorial Sloan Kettering	Low	• Description of surgery and radiotherapy	• No discussion of drug therapy • No references • No discussion about risk • No discussion about QoL
Squamous Cell Carcinoma	Moffitt Cancer Center	Low	• Surgical treatments well laid out	• No discussion of drug therapy • No references • No discussion about risk • No discussion about QoL
Skin Cancer Treatment—Patient Version	National Institutes of Health	Moderate	• Good descriptions of each treatment • Additional signposts • Patient encouraged to ask questions and seek a second opinion	• Side effects are not related to specific treatments • No discussion about QoL • No discussion about shared decision-making
Types of Skin Cancer	NYU Langone	Moderate	• Description of surgery and radiotherapy • Some discussion of treatment benefits	• No immunotherapy information • No discussion about QoL • No discussion about shared decision-making
Advanced Squamous Cell Carcinoma Treatment	Skin Cancer Foundation	Low	• Various treatment options are discussed	• Treatment benefits and risks are not discussed with detail • Areas of uncertainty are not discussed • References are not provided • No discussion about QoL
Squamous Cell Skin Cancer	Wikipedia	Moderate	• Relevant content • Well balanced • Treatment details included	• No discussion about QoL • No discussion about uncertainty • No discussion about shared decision-making

Outside of the 240 results, there were an additional 88 advertisements, some of which were duplicated either as repeat ads (53/60.2%) or within the main search results without being promoted. These ads included cancer centers, cancer charities (such as the Teenage Cancer Trust), private physician pages and other health-based websites. Some of this information was pertinent to treatment for melanoma or head and neck cancer, an overlap often seen with squamous cell carcinomas.

## Discussion and conclusion

### Discussion

This review demonstrates a lack of online information available for patients with high-risk or advanced cSCC. Information that is available is not always up to date and does not necessarily provide sufficient focus on the advanced setting. Given the overall absence of signposting to reliable information available to these patients, it is concerning that the disease profile was not prominent even within repositories that have become trusted sources of information.

This process has demonstrated the trade-off that patients face when searching for information about their condition. The broader search terms returned a wide array of information, but this was often lacking focus for the advanced or high-risk setting. However, more specific searches returned far too technical information. Searches 1–4 returned a large number of academic and clinical resources, particularly when using ‘advanced’ or ‘high risk’ terminology. Due to health literacy levels within the population, these are not necessarily appropriate resources. In fact, as a primary source of information available, these could be harmful or fear provoking due to the discussion around morbidity and mortality [38]. Patients will often use multiple websites to corroborate information, but they would need to use a mix of broad and targeted terms, as demonstrated here, for a better picture of the high-risk and advanced landscape [17].

A further area of confusion is the ambiguous language used when referring to high-risk and advanced disease. The words used, and their accompanying descriptions, differed by site. Those sites that are recognizable to patients, e.g. Macmillan and CRUK, did not use the terms at all. This may leave patients feeling unsure about the information they have previously received, either from their medical team or other websites.

This ambiguity is further compounded for UK patients as the majority of information was aimed at a US audience. While the information is still broadly relevant for patients globally, there are some nuances which are important for a UK setting. For example, conversations about insurance and care pathways do not necessarily translate. Additionally, the approach to care carries cultural nuance which may come across as inappropriate [38]. A prominent area of difference is that of the initial referral and then later care pathways. Patients within the UK may follow a different referral pathway as opposed to a patient within a private care system. Due to the lack of UK specific sources, these differences were poorly outlined. Research suggests non-metastatic patients struggle with informational needs with very little attention paid to the metastatic or high-risk setting [10]. Understanding the care pathway is one such area where patients need information and signposting.

The range of discern scores demonstrates the variability in the quality of information available. The majority of pages did not provide high quality information for patients to then use to make informed decisions. While this tool does not allow for assessments of accuracy, we did not find that any pages were providing biased or inaccurate information. Our primary concern was the omission of detail. For example, while all sites had been updated within the past 5 years to meet our inclusion criteria, they did not all contain the most current information about treatment, namely advances in immunotherapy. There were also few references to the impact treatments may have on quality of life, an area that is rarely addressed in the literature but so pertinent for patient decision-making [10].

#### Limitations

Inherent to this type of study is that searches will change over time, and our results will differ depending on individual search history and geographic location. However, by adding a repeat search run by a second researcher, we aim to present results which are representative of those a patient may find. That variability increases with broader search terms so these returns are more susceptible to variance. Future studies could replicate this form of methodology with input from patient groups as to the most used search terms. Different search engines could also be used, e.g. Bing, again in line with patient preference.

### Conclusion

The searches presented here demonstrate a need to ensure online information for patients with either high-risk or advanced cSCC is accessible, robust, accurate and written in patient friendly language. This patient group represents a smaller cohort amongst the cancer population but their informational needs are not negligible. With variability on terminology, care pathways and relevance, patients may be left with more questions than answers. In an age where more and more individuals with cancer turn to the Internet for additional support, further developmental work is needed to tailor appropriate information resources and target this directly to patients.

### Practice implications

To address this gap, further work could be undertaken by both by the clinical teams and charitable groups. Teams could signpost patients to reliable online information that contains the appropriate detail about the advanced or high-risk setting. While we found hospital-based information, this often only alluded to disease progression without further details. These resources could be expanded upon and made available online. Teams should also be aware of what information is currently available to patients as a way of pre-empting questions which may arise. There may be confusion, for example, with melanoma and how treatments differ. By understanding the current online landscape, clinics could then equip patients with key terms to search for or avoid.

Further work could also be undertaken by websites to ensure their information is current with clear guidance on pathways and treatments.

A better understanding of the patient experience in this setting, via qualitative methods, would provide a lived experience understanding of how best to produce materials and what information is needed.

## Data Availability

Not applicable

## References

[1] Nasser N, Nasser Filho N, Lehmkuhl RL (2015). Squamous cell cancer--31-year epidemiological study in a city of south Brazil. An Bras Dermatol.

[2] Apalla Z, Lallas A, Sotiriou E, Lazaridou E, Ioannides D (2017). Epidemiological trends in skin cancer. Dermatol pract & conceptual.

[3] Corchado-Cobos R, García-Sancha N, González-Sarmiento R, Pérez-Losada J, Cañueto J (2020). Cutaneous squamous cell carcinoma: from biology to therapy. Int J Mol Sci.

[4] Tanese K, Nakamura Y, Hirai I, Funakoshi T (2019) Updates on the systemic treatment of advanced non-melanoma skin cancer. Front in Med 6(160). 10.3389/fmed.2019.00160 10.3389/fmed.2019.00160 PMC6635480 31355203

[5] Bates AS, Davis CR, Takwale A, Knepil GJ (2013). Patient-reported outcome measures in nonmelanoma skin cancer of the face: a systematic review. Br J Dermatol.

[6] Migden MR, Rischin D, Schmults CD, Guminski A, Hauschild A, Lewis KD, Chung CH, Hernandez-Aya L, Lim AM, Chang ALS, Rabinowits G, Thai AA, Dunn LA, Hughes BGM, Khushalani NI, Modi B, Schadendorf D, Gao B, Seebach F, Li S, Li J, Mathias M, Booth J, Mohan K, Stankevich E, Babiker HM, Brana I, Gil-Martin M, Homsi J, Johnson ML, Moreno V, Niu J, Owonikoko TK, Papadopoulos KP, Yancopoulos GD, Lowy I, Fury MG (2018). PD-1 blockade with cemiplimab in advanced cutaneous squamous-cell carcinoma. N Engl J Med.

[7] Ogata D, Namikawa K, Otsuka M, Asai J, Kato H, Yasuda M, Maekawa T, Fujimura T, Kato J, Takenouchi T, Nagase K, Kawaguchi M, Kaji T, Kuwatsuka Y, Shibayama Y, Takai T, Okumura M, Kambayashi Y, Yoshikawa S, Yamazaki N, Tsuchida T (2020). Systemic treatment of patients with advanced cutaneous squamous cell carcinoma: response rates and outcomes of the regimes used. Eur J Cancer.

[8] Stratigos A, Garbe C, Lebbe C, Malvehy J, del Marmol V, Pehamberger H, Peris K, Becker JC, Zalaudek I, Saiag P, Middleton MR, Bastholt L, Testori A, Grob J-J (2015). Diagnosis and treatment of invasive squamous cell carcinoma of the skin: European consensus-based interdisciplinary guideline. Eur J Cancer.

[9] Jennings L, Schmults CD (2010). Management of high-risk cutaneous squamous cell carcinoma. J Clin Aesthet Dermatol.

[10] Körner A, Garland R, Czajkowska Z, Coroiu A, Khanna M (2016). Supportive care needs and distress in patients with non-melanoma skin cancer: nothing to worry about?. Eur J Oncol Nurs.

[11] Caddick J, Green L, Stephenson J, Spyrou G (2012). The psycho-social impact of facial skin cancers. J Plast Reconstr Aesthet Surg.

[12] Lange L, Peikert ML, Bleich C, Schulz H (2019). The extent to which cancer patients trust in cancer-related online information: a systematic review. PeerJ.

[13] Rick Waalboer-Spuij LMH, Nijsten TEC, van de Poll-Franse LV (2019). Satisfaction with information provision and health-related quality of life in basal and squamous cell carcinoma patients: a cross-sectional population-based study. Acta Derm Venereol.

[14] Motley R, Kersey P, Lawrence C (2002). Multiprofessional guidelines for the management of the patient with primary cutaneous squamous cell carcinoma. Br J Dermatol.

[15] Roberts N, Czajkowska Z, Radiotis G, Körner A (2013). Distress and coping strategies among patients with skin cancer. J Clin Psychol Med Settings.

[16] Winterbottom A, Harcourt D (2004). Patients’ experience of the diagnosis and treatment of skin cancer. J Adv Nurs.

[17] Maddock C, Lewis I, Ahmad K, Sullivan R (2011). Online information needs of cancer patients and their organizations. Ecancermedicalscience.

[18] Tan SS-L, Goonawardene N (2017). Internet health information seeking and the patient-physician relationship: a systematic review. J Med Internet Res.

[19] Tonsaker T, Bartlett G, Trpkov C (2014). Health information on the Internet: gold mine or minefield?. Can Fam Physician.

[20] Weeks N, McDonald FEJ, Patterson P, Konings S, Coad J (2019). A summary of high quality online information resources for parents with cancer who have adolescent and young adult children: a scoping review. Psychooncology.

[21] Jansen BJ, Spink A (2006). How are we searching the World Wide Web? A comparison of nine search engine transaction logs. Inf Process Manag.

[22] Charnock D, Shepperd S, Needham G, Gann R (1999). DISCERN: an instrument for judging the quality of written consumer health information on treatment choices. J Epidemiol Community Health.

[23] American Academy of Dermatology Association (2020) Squamous cell carcinoma: overview. https://www.aad.org/public/diseases/skin-cancer/types/common/scc. 2020

[24] Skin Cancer Foundation (2019) Advanced squamous cell carcinoma treatment. https://www.skincancer.org/skin-cancer-information/squamous-cell-carcinoma/advanced-scc/. 2020

[25] National Cancer Institute (2020) Skin cancer treatment (PDQ) - patient version. https://www.cancer.gov/types/skin/patient/skin-treatment-pdq. 2020

[26] Macmillan (2020) Skin Cancer. https://www.macmillan.org.uk/cancer-information-and-support/skin-cancer. 2020

[27] American Cancer Society (2019) Basal and squamous cell skin carcinoma. https://www.cancer.org/cancer/basal-and-squamous-cell-skin-cancer.html. 2020

[28] Cancer Research UK (2019) Skin cancer. https://www.cancerresearchuk.org/about-cancer/skin-cancer. 2020

[29] Moffitt Cancer Center (2018) Squamous cell carcinoma information. https://moffitt.org/cancers/squamous-cell-carcinoma/. 2020

[30] Memorial Sloan Kettering Cancer Center (2020) Squamous cell carcinoma. https://www.mskcc.org/cancer-care/types/squamous-cell-carcinoma. 2020

[31] Mayo Clinic (2019) Squamous cell carcinoma of the skin. https://www.mayoclinic.org/diseases-conditions/squamous-cell-carcinoma/symptoms-causes/syc-20352480. 2020

[32] NYU Langone Health (2020) Types of skin cancer. https://nyulangone.org/conditions/basal-squamous-cell-skin-cancers-in-adults/types. 2020

[33] The Christie NHS Foundation Trust (2015) Squamous cell carcinoma. https://www.christie.nhs.uk/patients-and-visitors/your-treatment-and-care/types-of-cancer/squamous-cell-carcinoma. 2020

[34] ASCO (2019) Skin cancer (non-melanoma). https://www.cancer.net/cancer-types/skin-cancer-non-melanoma. 2020

[35] Healthline (2020) Navigating life with advanced CSCC. https://www.healthline.com/program/navigating-life-with-advanced-cscc. 2020

[36] DermNet NZ (2015) Cutaneous squamous cell carcinoma. https://dermnetnz.org/topics/cutaneous-squamous-cell-carcinoma/. 2020

[37] Wikipedia (2020) Squamous cell skin cancer. https://en.wikipedia.org/wiki/Squamous_cell_skin_cancer. 2020

[38] Ziebland S, Chapple A, Dumelow C, Evans J, Prinjha S, Rozmovits L (2004). How the Internet affects patients’ experience of cancer: a qualitative study. BMJ.

